# Supplementary material for: Mendelian randomization reveals no correlations between herpesvirus infection and idiopathic pulmonary fibrosis
Source: PLoS One. 2023 Nov 28;18(11):e0295082. doi: 10.1371/journal.pone.0295082 (PMC10683991; doi:10.1371/journal.pone.0295082)
Supplement: S2 Fig — (DOCX) [file pone.0295082.s002.docx]

**
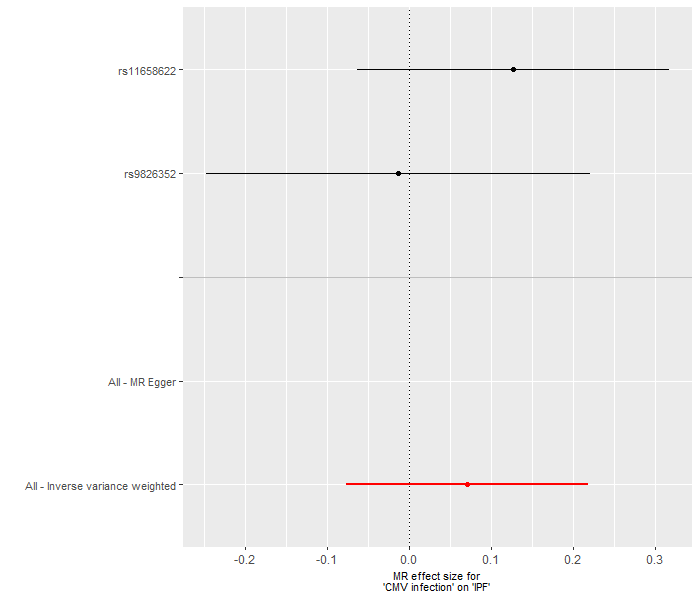
**

**
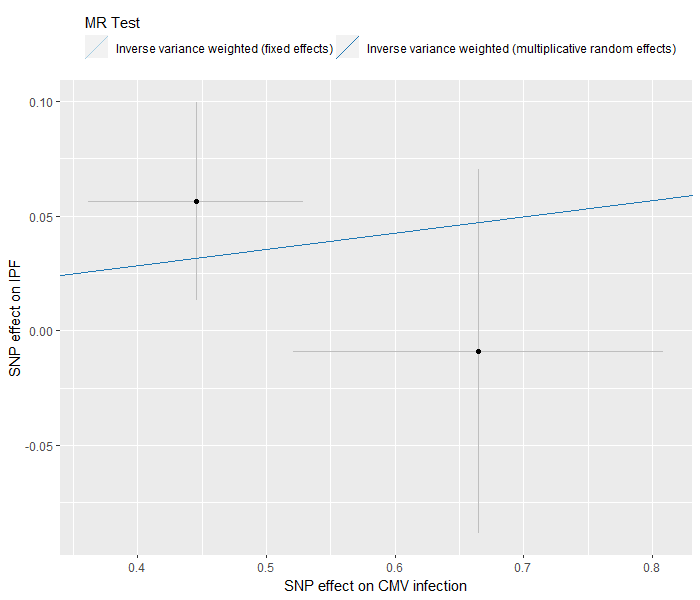
**

**S2 Fig.** The forest plot, and scatter plot for the association of CMV infection and idiopathic pulmonary fibrosis.
