# Supplementary material for: Mendelian randomization reveals no correlations between herpesvirus infection and idiopathic pulmonary fibrosis
Source: PLoS One. 2023 Nov 28;18(11):e0295082. doi: 10.1371/journal.pone.0295082 (PMC10683991; doi:10.1371/journal.pone.0295082)
Supplement: S5 Fig — (DOCX) [file pone.0295082.s005.docx]

**
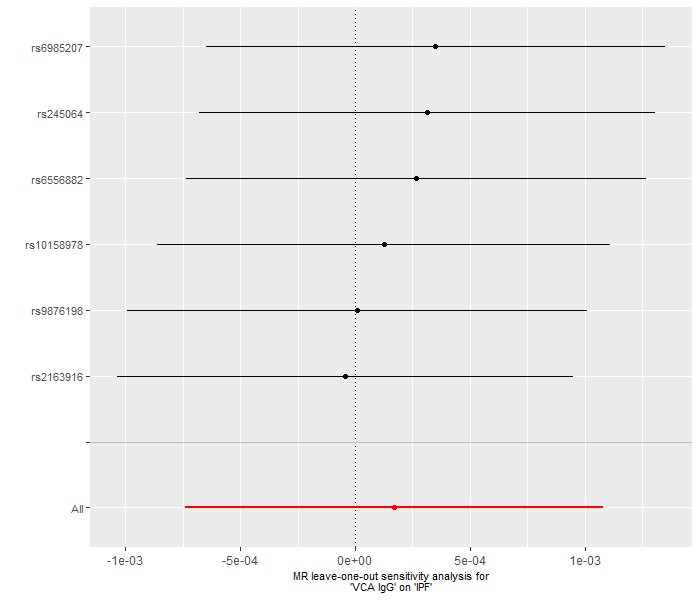
**

**
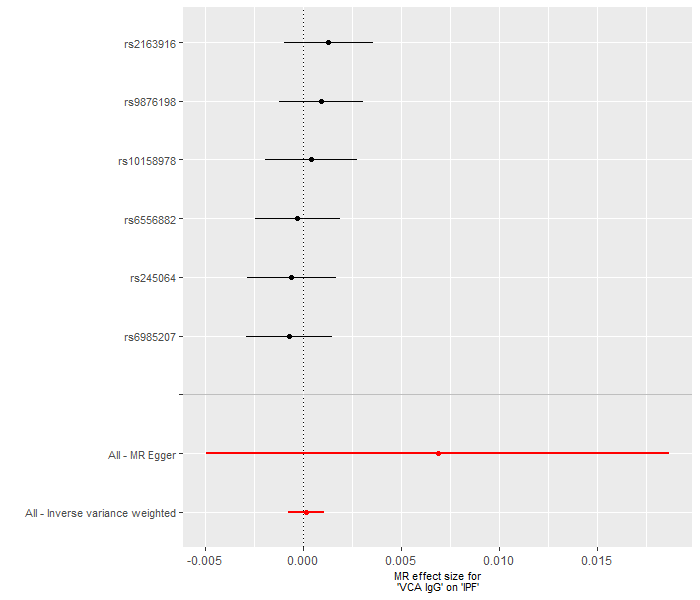
**

**
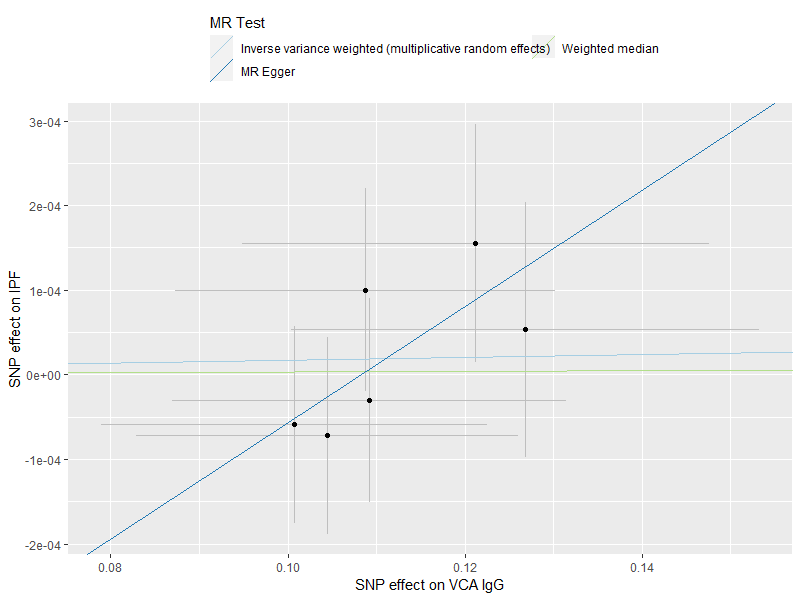
**

**S5 Fig.** The leave-one-out plot, forest plot, and scatter plot for the association of VCA IgG level and idiopathic pulmonary fibrosis in discovery analysis.
