# Supplementary material for: Mendelian randomization reveals no correlations between herpesvirus infection and idiopathic pulmonary fibrosis
Source: PLoS One. 2023 Nov 28;18(11):e0295082. doi: 10.1371/journal.pone.0295082 (PMC10683991; doi:10.1371/journal.pone.0295082)
Supplement: S9 Fig — (DOCX) [file pone.0295082.s009.docx]

**
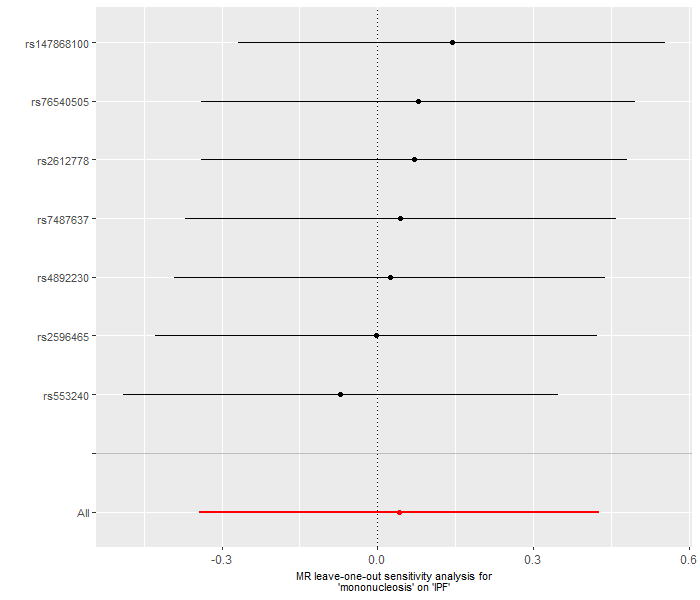

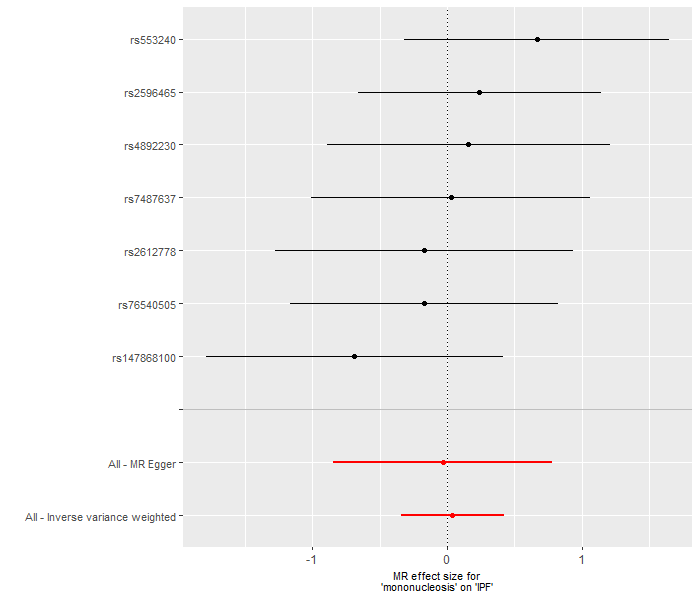

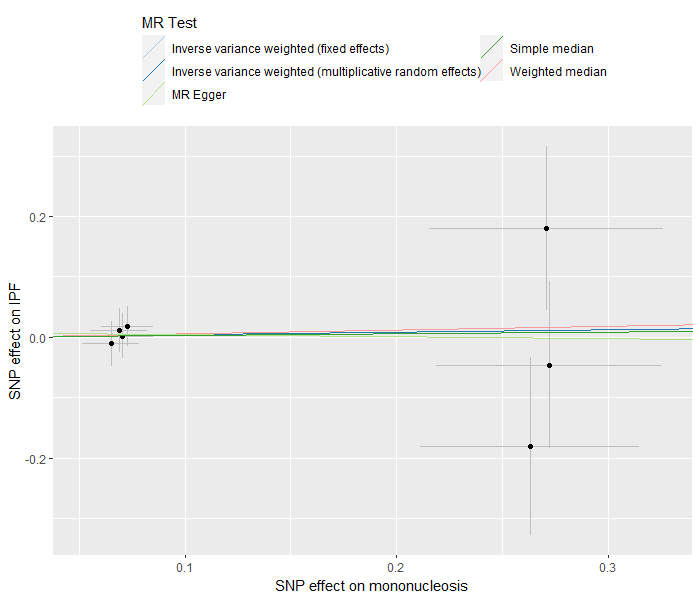
**

**S9 Fig.** The leave-one-out plot, forest plot, and scatter plot for the association of mononucleosis and idiopathic pulmonary fibrosis.
