# Supplementary material for: Mendelian randomization reveals no correlations between herpesvirus infection and idiopathic pulmonary fibrosis
Source: PLoS One. 2023 Nov 28;18(11):e0295082. doi: 10.1371/journal.pone.0295082 (PMC10683991; doi:10.1371/journal.pone.0295082)
Supplement: S1 Table — (DOCX) [file pone.0295082.s011.docx]

| **S1 Table. Brief description of datasets utilized in the Mendelian randomization study.** | | | | | | |
| --- | --- | --- | --- | --- | --- | --- |
| Exposure and **Outcome** | Source | Sample size | Cases | Controls | Population | Access Link |
| EBV infection | FinnGen cohort | 335,814 | 2,099 | 333,715 | European | <https://r8.finngen.fi/> |
| CMV infection |  | 301,867 | 428 | 301,439 | European | <https://r7.finngen.fi/> |
| HSV infection |  | 333,011 | 2,924 | 330,087 | European | <https://r8.finngen.fi/> |
| EBNA1 IgG | Milieu Intérieur cohort | 1,000 | 914 | 86 | European | <https://doi.org/10.5281/zenodo.1217136> |
| VCA IgG |  | 1,000 | 956 | 44 | European |  |
| CMV IgG |  | 1,000 | 347 | 653 | European |  |
| HSV-1 IgG |  | 1,000 | 645 | 355 | European |  |
| HSV-2 IgG |  | 1,000 | 208 | 792 | European |  |
| Mononucleosis | 23andMe cohort | 85,903 | 17,457 | 68,446 | European | PMID: 28928442 |
| Cold scores |  | 88,440 | 25,108 | 63,332 | European |  |
| **Idiopathic pulmonary fibrosis** | PMID: 31710517 | 11,259 | 2,668 | 8,591 | European | <https://github.com/genomicsITER/Pfgenetics> |
| Abbreviations: EBV, Epstein-Barr virus; CMV, cytomegalovirus; HSV, herpes simplex; EBNA1, EBV nuclear antigen-1; VCA, EBV viral capsid antigen; IgG, immunoglobulin G. | | | | | | |
