# Supplementary material for: Mendelian randomization reveals no correlations between herpesvirus infection and idiopathic pulmonary fibrosis
Source: PLoS One. 2023 Nov 28;18(11):e0295082. doi: 10.1371/journal.pone.0295082 (PMC10683991; doi:10.1371/journal.pone.0295082)
Supplement: S2 Table — (DOCX) [file pone.0295082.s012.docx]

| **S2 Table. Association of potential pleiotropic SNPs searched in the Phenoscanner database.** | | | | | | | |
| --- | --- | --- | --- | --- | --- | --- | --- |
| Exposure | SNP | Trait | Effect allele | Non-effect allele | Beta | P-value | PMID |
| HSV-1 | rs3134605 | Self-reported hypothyroidism or myxoedema | T | C | -0.0039 | 3.54E-10 | UKBB |
| EBNA1 | rs7452864 | Self-reported hypothyroidism or myxoedema | C | T | 0.0043 | 2.61E-13 | UKBB |
|  | rs7745002 | Self-reported hypothyroidism or myxoedema | A | G | -0.0069 | 8.42E-38 | UKBB |
|  | rs74951723 | Self-reported hypothyroidism or myxoedema | A | T | -0.006 | 2.18E-10 | UKBB |
| Abbreviations: SNP, single-nucleotide polymorphism; HSV, herpes simplex; EBNA1, EBV Epstein-Barr virus nuclear antigen-1. | | | | | | | |
