# Supplementary material for: Mendelian randomization reveals no correlations between herpesvirus infection and idiopathic pulmonary fibrosis
Source: PLoS One. 2023 Nov 28;18(11):e0295082. doi: 10.1371/journal.pone.0295082 (PMC10683991; doi:10.1371/journal.pone.0295082)
Supplement: S3 Table — (DOCX) [file pone.0295082.s013.docx]

| **S3 Table. Statistical power for the Mendelian randomization analyses of herpesvirus infection or herpesvirus infection-related IgG level and risk of IPF.** | | | | | |
| --- | --- | --- | --- | --- | --- |
| Exposure | Outcome | N SNPs | R^2^ | Power to identify OR  of 0.80 or 1.20 | Power to identify OR  of 0.70 or 1.45 |
| EBV infection | IPF | 13 | 0.145 | 1.170 | 1.150 |
| CMV infection | IPF | 2 | 0.110 | 1.194 | 1.172 |
| HSV infection | IPF | 7 | 0.052 | 1.288 | 1.255 |
| EBNA1 IgG | IPF | 6 | 0.135 | 1.176 | 1.155 |
| VCA IgG | IPF | 6 | 0.136 | 1.175 | 1.154 |
| CMV IgG | IPF | 16 | 0.398 | 1.101 | 1.089 |
| HSV-1 IgG | IPF | 3 | 0.070 | 1.248 | 1.219 |
| HSV-2 IgG | IPF | 8 | 0.197 | 1.144 | 1.128 |
| Mononucleosis | IPF | 7 | 0.011 | 1.660 | 1.580 |
| Cold scores | IPF | 6 | 0.007 | 1.850 | 1.750 |
| R^2^, proportion of variance explained for the association between the SNPs and the exposure variable. Abbreviations: SNPs: single nucleotide polymorphisms; se: standard error; IPF, idiopathic pulmonary fibrosis; EBV, Epstein-Barr virus; CMV, cytomegalovirus; HSV, herpes simplex; EBNA1, EBV nuclear antigen-1; VCA, EBV viral capsid antigen; IgG, immunoglobulin G. | | | | | |
