# Supplementary material for: Mendelian randomization reveals no correlations between herpesvirus infection and idiopathic pulmonary fibrosis
Source: PLoS One. 2023 Nov 28;18(11):e0295082. doi: 10.1371/journal.pone.0295082 (PMC10683991; doi:10.1371/journal.pone.0295082)
Supplement: S4 Table — (DOCX) [file pone.0295082.s014.docx]

| **S4 Table. Instrumental variables for genetically predicted herpesviruses infection or herpesvirus infection-related IgG level in the Mendelian randomization analysis.** | | | | | | |
| --- | --- | --- | --- | --- | --- | --- |
| Exposure | SNP | Effect  allele | Non-effect  allele | Effect allele  frequency | R^2^ | F |
|  |  |  |  |  |  |  |
| EBV infection | rs10797066 | C | T | 0.509 | 0.010 | 20.92 |
| EBV infection | rs138788751 | G | A | 0.012 | 0.010 | 21.06 |
| EBV infection | rs144973910 | A | G | 0.028 | 0.011 | 22.82 |
| EBV infection | rs17160581 | T | C | 0.094 | 0.010 | 21.57 |
| EBV infection | rs17578029 | A | T | 0.071 | 0.011 | 22.82 |
| EBV infection | rs192741093 | A | G | 0.024 | 0.012 | 26.08 |
| EBV infection | rs2794320 | G | A | 0.716 | 0.011 | 22.44 |
| EBV infection | rs318491 | G | A | 0.557 | 0.016 | 33.67 |
| EBV infection | rs73166878 | G | A | 0.061 | 0.011 | 22.74 |
| EBV infection | rs77967371 | A | G | 0.064 | 0.010 | 22.20 |
| EBV infection | rs906330 | C | T | 0.679 | 0.010 | 20.94 |
| EBV infection | rs9509967 | T | G | 0.129 | 0.012 | 26.27 |
| EBV infection | rs9922412 | T | C | 0.493 | 0.012 | 24.47 |
| CMV infection | rs11658622 | A | C | 0.760 | 0.062 | 28.40 |
| CMV infection | rs9826352 | G | C | 0.036 | 0.047 | 21.17 |
| HSV infection | rs117528127 | C | T | 0.018 | 0.008 | 22.66 |
| HSV infection | rs13160891 | T | C | 0.209 | 0.008 | 22.74 |
| HSV infection | rs17015920 | C | T | 0.043 | 0.008 | 22.41 |
| HSV infection | rs4325176 | G | A | 0.053 | 0.008 | 22.68 |
| HSV infection | rs4716482 | C | A | 0.519 | 0.007 | 21.03 |
| HSV infection | rs7252670 | G | T | 0.331 | 0.007 | 21.41 |
| HSV infection | rs72831240 | T | C | 0.203 | 0.007 | 21.21 |
| EBNA1 IgG | rs530411 | T | C | 0.347 | 0.025 | 25.44 |
| EBNA1 IgG | rs10226349 | T | C | 0.086 | 0.023 | 23.70 |
| EBNA1 IgG | rs4555924 | G | A | 0.133 | 0.023 | 23.18 |
| EBNA1 IgG | rs59217282 | T | C | 0.118 | 0.022 | 22.12 |
| EBNA1 IgG | rs17452718 | G | T | 0.134 | 0.021 | 21.52 |
| EBNA1 IgG | rs6895504 | C | T | 0.206 | 0.021 | 21.42 |
| VCA IgG | rs10158978 | A | T | 0.192 | 0.023 | 22.98 |
| VCA IgG | rs9876198 | T | C | 0.391 | 0.025 | 25.57 |
| VCA IgG | rs6556882 | C | T | 0.370 | 0.024 | 24.12 |
| VCA IgG | rs245064 | T | C | 0.426 | 0.021 | 21.29 |
| VCA IgG | rs6985207 | C | A | 0.435 | 0.023 | 23.42 |
| VCA IgG | rs2163916 | A | G | 0.209 | 0.021 | 21.12 |
| CMV IgG | rs7583185 | G | A | 0.060 | 0.027 | 27.37 |
| CMV IgG | rs77726835 | T | A | 0.079 | 0.024 | 24.05 |
| CMV IgG | rs1928191 | C | A | 0.121 | 0.021 | 21.90 |
| CMV IgG | rs113033410 | T | C | 0.169 | 0.025 | 25.35 |
| CMV IgG | rs1001036 | T | G | 0.055 | 0.032 | 32.81 |
| CMV IgG | rs16929628 | A | G | 0.041 | 0.023 | 23.99 |
| CMV IgG | rs1600519 | A | C | 0.111 | 0.033 | 33.80 |
| CMV IgG | rs76825464 | A | T | 0.057 | 0.024 | 24.71 |
| CMV IgG | rs79686415 | T | C | 0.070 | 0.023 | 23.81 |
| CMV IgG | rs77577412 | A | C | 0.052 | 0.023 | 23.77 |
| CMV IgG | rs4899627 | G | A | 0.045 | 0.022 | 21.96 |
| CMV IgG | rs35701456 | C | A | 0.036 | 0.029 | 30.16 |
| CMV IgG | rs72862405 | A | G | 0.066 | 0.021 | 21.88 |
| CMV IgG | rs76027104 | G | C | 0.048 | 0.022 | 22.15 |
| CMV IgG | rs58607100 | A | C | 0.172 | 0.025 | 25.44 |
| HSV-1 IgG | rs1738233 | T | A | 0.426 | 0.023 | 23.66 |
| HSV-1 IgG | rs58599785 | T | C | 0.166 | 0.021 | 21.23 |
| HSV-1 IgG | rs10977313 | T | G | 0.107 | 0.026 | 26.85 |
| HSV-2 IgG | rs10888851 | G | C | 0.109 | 0.023 | 23.15 |
| HSV-2 IgG | rs10782620 | G | T | 0.397 | 0.023 | 23.65 |
| HSV-2 IgG | rs10174926 | C | T | 0.126 | 0.025 | 25.83 |
| HSV-2 IgG | rs72804080 | G | A | 0.129 | 0.029 | 29.48 |
| HSV-2 IgG | rs355547 | C | T | 0.388 | 0.024 | 24.23 |
| HSV-2 IgG | rs35213774 | G | A | 0.112 | 0.025 | 25.55 |
| HSV-2 IgG | rs10964023 | T | G | 0.190 | 0.022 | 22.96 |
| HSV-2 IgG | rs10790877 | A | G | 0.474 | 0.026 | 26.32 |
| Mononucleosis | rs147868100 | A | G | 0.013 | 0.001 | 25.88 |
| Mononucleosis | rs2596465 | C | T | 0.554 | 0.002 | 35.70 |
| Mononucleosis | rs2612778 | C | T | 0.716 | 0.001 | 22.91 |
| Mononucleosis | rs4892230 | A | G | 0.286 | 0.001 | 26.12 |
| Mononucleosis | rs553240 | C | T | 0.985 | 0.001 | 23.94 |
| Mononucleosis | rs7487637 | A | G | 0.278 | 0.001 | 24.03 |
| Mononucleosis | rs76540505 | A | G | 0.015 | 0.002 | 26.32 |
| Cold scores | rs115789906 | G | T | 0.019 | 0.001 | 21.55 |
| Cold scores | rs16974161 | A | G | 0.035 | 0.001 | 24.20 |
| Cold scores | rs17732209 | C | T | 0.231 | 0.001 | 21.63 |
| Cold scores | rs73036068 | C | T | 0.031 | 0.001 | 26.66 |
| Cold scores | rs885950 | A | C | 0.438 | 0.002 | 51.47 |
| Cold scores | rs9266276 | A | G | 0.274 | 0.002 | 41.83 |
| Abbreviations: SNP, single-nucleotide polymorphism; R^2^, percentage of the variation of coffee consumption explained by the SNP; F, F statistic; Beta, the per-allele effect on coffee consumption; SE, Standard Error; P-value, the value for the genetic association; EBV, Epstein-Barr virus; CMV, cytomegalovirus; HSV, herpes simplex; EBNA1, Epstein-Barr virus nuclear antigen-1; VCA, EBV viral capsid antigen; IgG, immunoglobulin G. | | | | | | |
