# Supplementary material for: Mendelian randomization reveals no correlations between herpesvirus infection and idiopathic pulmonary fibrosis
Source: PLoS One. 2023 Nov 28;18(11):e0295082. doi: 10.1371/journal.pone.0295082 (PMC10683991; doi:10.1371/journal.pone.0295082)
Supplement: S5 Table — (DOCX) [file pone.0295082.s015.docx]

| **S5 Table. Summary information for SNPs that were used as genetic instruments for Mendelian randomization analyses of genetically predicted herpesviruses infection or herpesvirus-related IgG levels and risk of IPF.** | | | | | | | | | | |
| --- | --- | --- | --- | --- | --- | --- | --- | --- | --- | --- |
| Exposure | SNP | Effect  allele | Non-effect  allele | Effect allele  frequency | Beta | SE | P-value | IPF | | |
|  |  |  |  |  |  |  |  | Beta | SE | P-value |
| EBV infection | rs10797066 | C | T | 0.509 | -0.1421 | 0.0311 | 4.75E-06 | 0.0443 | 0.3222 | 0.891 |
| EBV infection | rs138788751 | G | A | 0.012 | -0.8499 | 0.1851 | 4.4E-06 | 0.0036 | 0.2498 | 0.988 |
| EBV infection | rs144973910 | A | G | 0.028 | 0.3991 | 0.0835 | 1.76E-06 | 0.0637 | 0.3418 | 0.852 |
| EBV infection | rs17160581 | T | C | 0.094 | 0.2332 | 0.0502 | 3.37E-06 | 0.1235 | 0.3379 | 0.715 |
| EBV infection | rs17578029 | A | T | 0.071 | 0.2699 | 0.0565 | 1.76E-06 | -0.3286 | 0.3353 | 0.327 |
| EBV infection | rs192741093 | A | G | 0.024 | -0.6239 | 0.1221 | 3.24E-07 | -0.0909 | 0.2370 | 0.701 |
| EBV infection | rs2794320 | G | A | 0.716 | -0.1602 | 0.0338 | 2.15E-06 | 0.2778 | 0.3165 | 0.380 |
| EBV infection | rs318491 | G | A | 0.557 | 0.1845 | 0.0318 | 6.41E-09 | -0.4939 | 0.2521 | 0.050 |
| EBV infection | rs73166878 | G | A | 0.061 | 0.2878 | 0.0603 | 1.84E-06 | -0.4667 | 0.3322 | 0.160 |
| EBV infection | rs77967371 | A | G | 0.064 | 0.2821 | 0.0598 | 2.43E-06 | -0.1570 | 0.3400 | 0.644 |
| EBV infection | rs906330 | C | T | 0.679 | 0.1551 | 0.0339 | 4.68E-06 | -0.3133 | 0.3159 | 0.321 |
| EBV infection | rs9509967 | T | G | 0.129 | -0.2511 | 0.0490 | 2.93E-07 | -0.2330 | 0.2728 | 0.393 |
| EBV infection | rs9922412 | T | C | 0.493 | 0.1535 | 0.0310 | 7.46E-07 | 0.2652 | 0.2971 | 0.372 |
| CMV infection | rs11658622 | A | C | 0.760 | 0.4453 | 0.0834 | 9.23E-08 | 0.1267 | 0.0969 | 0.191 |
| CMV infection | rs9826352 | G | C | 0.036 | 0.6649 | 0.1442 | 4.00E-06 | -0.0138 | 0.1193 | 0.908 |
| HSV infection | rs117528127 | C | T | 0.018 | -0.570 | 0.1196 | 1.91E-06 | 0.0764 | 0.3062 | 0.803 |
| HSV infection | rs13160891 | T | C | 0.209 | 0.149 | 0.0313 | 1.84E-06 | 0.2579 | 0.3744 | 0.491 |
| HSV infection | rs17015920 | C | T | 0.043 | -0.338 | 0.0713 | 2.19E-06 | -0.1638 | 0.3339 | 0.624 |
| HSV infection | rs4325176 | G | A | 0.053 | 0.261 | 0.0548 | 1.9E-06 | 0.0521 | 0.3945 | 0.895 |
| HSV infection | rs4716482 | C | A | 0.519 | -0.121 | 0.0263 | 4.49E-06 | -0.5358 | 0.3799 | 0.158 |
| HSV infection | rs7252670 | G | T | 0.331 | 0.127 | 0.0275 | 3.69E-06 | -0.2170 | 0.3829 | 0.571 |
| HSV infection | rs72831240 | T | C | 0.203 | -0.155 | 0.0337 | 4.08E-06 | 0.3461 | 0.3706 | 0.350 |
| EBNA1 IgG | rs530411 | T | C | 0.347 | 0.1399 | 0.0277 | 5.37E-07 | 0.0150 | 0.0351 | 0.669 |
| EBNA1 IgG | rs10226349 | T | C | 0.086 | 0.2252 | 0.0462 | 1.29E-06 | 0.0124 | 0.0598 | 0.835 |
| EBNA1 IgG | rs4555924 | G | A | 0.133 | 0.1903 | 0.0395 | 1.68E-06 | -0.0393 | 0.0492 | 0.424 |
| EBNA1 IgG | rs59217282 | T | C | 0.118 | -0.1933 | 0.0410 | 2.89E-06 | 0.0082 | 0.0530 | 0.878 |
| EBNA1 IgG | rs17452718 | G | T | 0.134 | -0.1776 | 0.0382 | 3.93E-06 | -0.0049 | 0.0488 | 0.920 |
| EBNA1 IgG | rs6895504 | C | T | 0.206 | 0.1476 | 0.0319 | 4.14E-06 | -0.0221 | 0.0408 | 0.588 |
| VCA IgG | rs10158978 | A | T | 0.192 | -0.1268 | 0.0264 | 1.86E-06 | -0.0495 | 0.0431 | 0.251 |
| VCA IgG | rs9876198 | T | C | 0.391 | -0.1088 | 0.0215 | 5.00E-07 | 0.0054 | 0.0347 | 0.876 |
| VCA IgG | rs6556882 | C | T | 0.370 | -0.1092 | 0.0222 | 1.04E-06 | 0.0149 | 0.0345 | 0.665 |
| VCA IgG | rs245064 | T | C | 0.426 | -0.1007 | 0.0218 | 4.39E-06 | 0.0130 | 0.0337 | 0.701 |
| VCA IgG | rs6985207 | C | A | 0.435 | -0.1044 | 0.0216 | 1.49E-06 | -0.0033 | 0.0409 | 0.936 |
| VCA IgG | rs2163916 | A | G | 0.209 | -0.1212 | 0.0263 | 4.78E-06 | -0.0334 | 0.0395 | 0.398 |
| CMV IgG | rs7583185 | G | A | 0.060 | -0.2184 | 0.0417 | 2.90E-07 | 0.0816 | 0.0736 | 0.268 |
| CMV IgG | rs77726835 | T | A | 0.079 | -0.1739 | 0.0354 | 1.43E-06 | -0.0530 | 0.0594 | 0.372 |
| CMV IgG | rs1928191 | C | A | 0.121 | -0.1390 | 0.0297 | 4.09E-06 | 0.0139 | 0.0495 | 0.778 |
| CMV IgG | rs113033410 | T | C | 0.169 | -0.1358 | 0.0269 | 7.64E-07 | -0.0320 | 0.0470 | 0.496 |
| CMV IgG | rs1001036 | T | G | 0.055 | -0.2558 | 0.0446 | 2.20E-08 | -0.0216 | 0.0819 | 0.792 |
| CMV IgG | rs16929628 | A | G | 0.041 | -0.2414 | 0.0492 | 1.48E-06 | -0.1513 | 0.0782 | 0.053 |
| CMV IgG | rs1600519 | A | C | 0.111 | -0.1673 | 0.0287 | 1.38E-08 | 0.0160 | 0.0529 | 0.763 |
| CMV IgG | rs76825464 | A | T | 0.057 | -0.2212 | 0.0445 | 1.04E-06 | -0.0102 | 0.0805 | 0.899 |
| CMV IgG | rs79686415 | T | C | 0.070 | -0.1764 | 0.0361 | 1.61E-06 | -0.0167 | 0.0750 | 0.824 |
| CMV IgG | rs77577412 | A | C | 0.052 | -0.2171 | 0.0445 | 1.65E-06 | 0.1049 | 0.0751 | 0.163 |
| CMV IgG | rs4899627 | G | A | 0.045 | -0.2361 | 0.0503 | 3.97E-06 | -0.0261 | 0.0682 | 0.702 |
| CMV IgG | rs35701456 | C | A | 0.036 | -0.2724 | 0.0495 | 7.66E-08 | -0.0758 | 0.0805 | 0.346 |
| CMV IgG | rs72862405 | A | G | 0.066 | -0.1846 | 0.0394 | 4.13E-06 | -0.0512 | 0.0770 | 0.506 |
| CMV IgG | rs76027104 | G | C | 0.048 | -0.2242 | 0.0476 | 3.62E-06 | -0.0711 | 0.0679 | 0.295 |
| CMV IgG | rs58607100 | A | C | 0.172 | -0.1274 | 0.0252 | 7.31E-07 | -0.0304 | 0.0459 | 0.509 |
| HSV-1 IgG | rs1738233 | T | A | 0.426 | 0.0724 | 0.0149 | 1.45E-06 | -0.0136 | 0.0339 | 0.689 |
| HSV-1 IgG | rs58599785 | T | C | 0.166 | 0.0865 | 0.0188 | 4.91E-06 | 0.0390 | 0.0459 | 0.395 |
| HSV-1 IgG | rs10977313 | T | G | 0.107 | -0.1253 | 0.0241 | 2.97E-07 | 0.0093 | 0.0513 | 0.856 |
| HSV-2 IgG | rs10888851 | G | C | 0.109 | -0.2401 | 0.0498 | 3.27E-06 | 0.0354 | 0.0512 | 0.490 |
| HSV-2 IgG | rs10782620 | G | T | 0.397 | 0.1628 | 0.0334 | 2.60E-06 | -0.0383 | 0.0334 | 0.252 |
| HSV-2 IgG | rs10174926 | C | T | 0.126 | -0.2364 | 0.0465 | 9.72E-07 | 0.0232 | 0.0541 | 0.668 |
| HSV-2 IgG | rs72804080 | G | A | 0.129 | 0.2591 | 0.0477 | 1.92E-07 | -0.0574 | 0.0477 | 0.229 |
| HSV-2 IgG | rs355547 | C | T | 0.388 | 0.1726 | 0.0350 | 2.00E-06 | -0.0526 | 0.0345 | 0.127 |
| HSV-2 IgG | rs35213774 | G | A | 0.112 | 0.2652 | 0.0524 | 1.10E-06 | -0.0288 | 0.0490 | 0.557 |
| HSV-2 IgG | rs10964023 | T | G | 0.190 | -0.1936 | 0.0404 | 3.58E-06 | -0.0431 | 0.0426 | 0.311 |
| HSV-2 IgG | rs10790877 | A | G | 0.474 | -0.1623 | 0.0316 | 7.82E-07 | 0.0427 | 0.0333 | 0.199 |
| Mononucleosis | rs147868100 | A | G | 0.013 | -0.263 | 0.0517 | 6.07E-07 | -0.687 | 0.562 | 0.222 |
| Mononucleosis | rs2596465 | C | T | 0.554 | 0.0729 | 0.0122 | 2.48E-09 | 0.242 | 0.462 | 0.600 |
| Mononucleosis | rs2612778 | C | T | 0.716 | -0.0651 | 0.0136 | 1.67E-06 | -0.168 | 0.564 | 0.766 |
| Mononucleosis | rs4892230 | A | G | 0.286 | -0.069 | 0.0135 | 3.22E-07 | 0.159 | 0.536 | 0.767 |
| Mononucleosis | rs553240 | C | T | 0.985 | -0.2706 | 0.0553 | 1.59E-06 | 0.666 | 0.502 | 0.184 |
| Mononucleosis | rs7487637 | A | G | 0.278 | 0.0706 | 0.0144 | 9.23E-07 | 0.027 | 0.529 | 0.959 |
| Mononucleosis | rs76540505 | A | G | 0.015 | 0.2719 | 0.053 | 5.17E-07 | -0.170 | 0.508 | 0.739 |
| Cold scores | rs115789906 | G | T | 0.019 | 0.195 | 0.042 | 2.52E-06 | 0.401 | 0.631 | 0.525 |
| Cold scores | rs16974161 | A | G | 0.035 | 0.1825 | 0.0371 | 1.02E-06 | -0.713 | 0.508 | 0.161 |
| Cold scores | rs17732209 | C | T | 0.231 | 0.0586 | 0.0126 | 3.79E-06 | -0.081 | 0.678 | 0.905 |
| Cold scores | rs73036068 | C | T | 0.031 | -0.2055 | 0.0398 | 3.00E-07 | 0.890 | 0.466 | 0.056 |
| Cold scores | rs885950 | A | C | 0.438 | 0.0782 | 0.0109 | 7.47E-13 | -0.388 | 0.434 | 0.371 |
| Cold scores | rs9266276 | A | G | 0.274 | -0.0815 | 0.0126 | 9.96E-11 | -0.513 | 0.463 | 0.268 |
| Abbreviations: SNP, single-nucleotide polymorphism; R^2^, percentage of the variation of coffee consumption explained by the SNP; F, F statistic; Beta, the per-allele effect on coffee consumption; SE, Standard Error; P-value, the value for the genetic association; EBV, Epstein-Barr virus; CMV, cytomegalovirus; HSV, herpes simplex; IPF, idiopathic pulmonary fibrosis; EBNA1, Epstein-Barr virus nuclear antigen-1; VCA, EBV viral capsid antigen; IgG, immunoglobulin G. | | | | | | | | | | |
