# Supplementary material for: Mendelian randomization reveals no correlations between herpesvirus infection and idiopathic pulmonary fibrosis
Source: PLoS One. 2023 Nov 28;18(11):e0295082. doi: 10.1371/journal.pone.0295082 (PMC10683991; doi:10.1371/journal.pone.0295082)
Supplement: S6 Table — (DOCX) [file pone.0295082.s016.docx]

| **S6 Table. Association of SNPs for herpesvirus infection or herpesvirus infection-related IgG level with IPF using MR with different methods.** | | | | | |
| --- | --- | --- | --- | --- | --- |
| Exposure | Outcome | SNPs,n | Methods | OR (95%CI) | P-value |
|  |  |  |  |  |  |
| EBV infection | IPF | 13 | IVW | 1.015(0.897–1.149) | 0.815 |
|  |  |  | WM | 1.028(0.866–1.219) | 0.753 |
|  |  |  | MR-Egger | 1.110(0.837–1.473) | 0.211 |
| CMV infection^1^ | IPF | 2 | IVW | 1.073(0.926–1.244) | 0.302 |
| HSV infection | IPF | 7 | IVW | 0.906(0.753–1.091) | 0.298 |
|  |  |  | WM | 0.926(0.716–1.198) | 0.560 |
|  |  |  | MR-Egger | 0.555(0.364–0.846) | 0.041 |
| EBNA1 IgG | IPF | 6 | IVW | 0.968(0.782–1.198) | 0.764 |
|  |  |  | WM | 0.999(0.768–1.300) | 0.995 |
|  |  |  | MR-Egger | 0.887(0.242–3.258) | 0.866 |
| VCA IgG | IPF | 6 | IVW | 1.061(0.811–1.387) | 0.665 |
|  |  |  | WM | 0.975(0.703–1.353) | 0.881 |
|  |  |  | MR-Egger | 10.995(0.356–339.710) | 0.243 |
| CMV IgG | IPF | 15 | IVW | 1.108(0.944–1.314) | 0.240 |
|  |  |  | WM | 1.105(0.868–1.407) | 0.419 |
|  |  |  | MR-Egger | 1.027(0.505–2.091) | 0.942 |
| HSV-1 IgG | IPF | 3 | IVW | 1.154(0.684–1.945) | 0.592 |
|  |  |  | WM | 1.132(0.606–2.117) | 0.697 |
|  |  |  | MR-Egger | 0.631(0.065–6.141) | 0.760 |
| HSV-2 IgG | IPF | 8 | IVW | 0.915(0.793–1.056) | 0.225 |
|  |  |  | WM | 0.874(0.722–1.058) | 0.167 |
|  |  |  | MR-Egger | 0.724(0.338–1.551) | 0.438 |
| Mononucleosis | IPF | 7 | IVW | 1.042(0.709–1.532) | 0.832 |
|  |  |  | WM | 1.064(0.642–1.762) | 0.810 |
|  |  |  | MR-Egger | 0.969(0.429–2.188) | 0.942 |
| Cold scores | IPF | 6 | IVW | 0.906(0.603–1.362) | 0.635 |
|  |  |  | WM | 0.695(0.403–1.199) | 0.191 |
|  |  |  | MR-Egger | 1.653(0.489–5.587) | 0.464 |
| ^1^The weighted median approach provide valid results even if some of the genetic variants are invalid. The MR-Egger method is the causal effect estimate adjusted for horizontal pleiotropy. Those estimates could not be calculated for CMV infection because there are less than three SNPs were available. Abbreviations: SNP, single-nucleotide polymorphism; OR, odds ratio; CI, confidence interval; SE, Standard Error; MR-PRESSO, Mendelian Randomization Pleiotropy RESidual Sum and Outlier; IVW, inverse variance weighted; WM, weighted median; IPF, idiopathic pulmonary fibrosis; EBV, Epstein-Barr virus; CMV, cytomegalovirus; HSV, herpes simplex; EBNA1, EBV nuclear antigen-1; VCA, EBV viral capsid antigen; IgG, immunoglobulin G. | | | | | |
|  |  |  |  |  |  |
