# Supplementary material for: Mendelian randomization reveals no correlations between herpesvirus infection and idiopathic pulmonary fibrosis
Source: PLoS One. 2023 Nov 28;18(11):e0295082. doi: 10.1371/journal.pone.0295082 (PMC10683991; doi:10.1371/journal.pone.0295082)
Supplement: S7 Table — (DOCX) [file pone.0295082.s017.docx]

| **S7 Table. Cochran’s Q test, MR-Egger intercept and MR-PRESSO Mendelian randomization analyses of herpesvirus infection or herpesvirus infection-related IgG level and risk of IPF.** | | | | | | | | | |
| --- | --- | --- | --- | --- | --- | --- | --- | --- | --- |
| Exposure | Outcome | SNPs,n | Heterogeneity | | Pleiotropy | | | MR-PRESSO | |
|  |  |  | Cochran’s Q  statistic^1^ | P-value | MR-Egger intercept^2^ | se | P-value | Global Test^3^ | P-value |
| EBV infection | IPF | 13 | 8.543 | 0.741 | -0.0234 | 0.0337 | 0.502 | 10.463 | 0.731 |
| CMV infection | IPF | 2 | 0.835 | 0.361 | - | - | - | - | - |
| HSV infection | IPF | 7 | 11.556 | 0.073 | 0.1006 | 0.0396 | 0.052 | 15.338 | 0.108 |
| EBNA1 IgG | IPF | 6 | 1.102 | 0.954 | 0.0151 | 0.1137 | 0.901 | 1.618 | 0.971 |
| VCA IgG | IPF | 6 | 2.210 | 0.819 | -0.2604 | 0.1944 | 0.251 | 3.219 | 0.812 |
| CMV IgG | IPF | 15 | 10.114 | 0.754 | 0.0146 | 0.0682 | 0.834 | 11.685 | 0.761 |
| HSV-1 IgG | IPF | 3 | 0.627 | 0.731 | 0.0560 | 0.1049 | 0.688 | - | - |
| HSV-2 IgG | IPF | 8 | 7.238 | 0.405 | 0.0480 | 0.0782 | 0.562 | 9.392 | 0.430 |
| Mononucleosis | IPF | 7 | 3.775 | 0.707 | 0.0075 | 0.0372 | 0.849 | 6.736 | 0.653 |
| Cold scores | IPF | 6 | 7.842 | 0.165 | -0.0636 | 0.0598 | 0.348 | 11.497 | 0.250 |
| ^1^The Cochran’s Q test is a statistical test for heterogeneity.  ^2^The intercept term from the MR-Egger regression method is a statistical test of horizontal pleiotropy. This statistic could not be calculated for CMV infection because less than three SNPs were available. ^3^The MR-PRESSO method detected the existence of outlier IVs that may have horizontal pleiotropy through the global test. This statistic could not be calculated for CMV infection and HSV-1 IgG because less than four SNPs were available. Abbreviations: MR-PRESSO: the Mendelian Randomization Pleiotropy RESidual Sum and Outlier; SNPs: single nucleotide polymorphisms; se: standard error; IPF, idiopathic pulmonary fibrosis; EBV, Epstein-Barr virus; CMV, cytomegalovirus; HSV, herpes simplex; EBNA1, EBV nuclear antigen-1; VCA, EBV viral capsid antigen; IgG, immunoglobulin G. | | | | | | | | | |
