# Supplementary material for: Mendelian randomization reveals no correlations between herpesvirus infection and idiopathic pulmonary fibrosis
Source: PLoS One. 2023 Nov 28;18(11):e0295082. doi: 10.1371/journal.pone.0295082 (PMC10683991; doi:10.1371/journal.pone.0295082)
Supplement: Attachment — Submitted filename: Response to Reviewers.docx [file pone.0295082.s018.docx]

**Reviewer 1**

Dear Reviewer,

Thanks very much for taking your time to review this manuscript. I really appreciate your comments and suggestions! Please see below, for a point-by-point response to the reviewer’s comments and concerns. I hope that the changes I’ve made resolve all your concerns about the article. I’m more than happy to make any further changes that will improve the paper and facilitate successful publication.

**Point 1:** The strength of association between the chosen SNPs, which serve as instrumental variables (IVs), and the exposures (i.e., herpes viral infections) remains unclear. A core premise of MR studies is the tight correlation between IVs and exposure. From the presented data, the selected IVs seem to exhibit a weak relationship with herpes infection/IgG levels. Should there be a strong association, one would expect to observe the same SNPs consistently identified across diverse study cohorts. However, no such overlap is evident among IVs from different cohorts, even though each has its distinct criteria for determining herpesvirus infection. Given the high prevalence of herpesvirus infections in humans, it seems improbable to pinpoint SNPs significantly associated with these infections.

**Response 1:** Thank you for pointing this out. Indeed, there is a relatively weak association between some selected IVs and herpesvirus infection/IgG levels. Considering the high prevalence of herpesvirus infection in humans, it is challenging to pinpoint SNPs significantly correlated with these infections. However, to address this limitation, we opted for three independent cohorts to validate the impact of herpesvirus infection on IPF onset, thereby enhancing the credibility of our results. It is worth noting that the herpesvirus infection-related data used in this study have been employed in several MR studies. This suggests that utilizing these data for herpesvirus-related MR analysis is reasonably justified to some extent. Specific papers for reference are as follows:

1. Zhang W, Wu P, Yin R, et al. Mendelian Randomization Analysis Suggests No Associations of Herpes Simplex Virus Infections With Multiple Sclerosis. Front Neurosci. 2022;16:817067. Published 2022 Mar 1. doi:10.3389/fnins.2022.817067.
2. Huang SY, Yang YX, Kuo K, et al. Herpesvirus infections and Alzheimer's disease: a Mendelian randomization study. Alzheimers Res Ther. 2021;13(1):158. Published 2021 Sep 24. doi:10.1186/s13195-021-00905-5.
3. Zhang Y, Qu J, Luo L, Xu Z, Zou X. Multigenomics Reveals the Causal Effect of Herpes Simplex Virus in Alzheimer's Disease: A Two-Sample Mendelian Randomization Study. Front Genet. 2022;12:773725. Published 2022 Jan 5. doi:10.3389/fgene.2021.773725.
4. Tan JS, Ren JM, Fan L, et al. Genetic Predisposition of Anti-Cytomegalovirus Immunoglobulin G Levels and the Risk of 9 Cardiovascular Diseases. Front Cell Infect Microbiol. 2022;12:884298. Published 2022 Jun 27. doi:10.3389/fcimb.2022.884298.
5. Zhu G, Zhou S, Xu Y, et al. Chickenpox and multiple sclerosis: A Mendelian randomization study. J Med Virol. 2023;95(1):e28315. doi:10.1002/jmv.28315.

**Point 2:** As the study concludes with a negative correlation, it might be beneficial to apply the same MR method to investigate a known positive risk factor for IPF, such as smoking. Demonstrating such a correlation would greatly bolster the credibility of the findings and enhance reader confidence.

**Response 2:** We have identified an existing MR study that investigated the causal relationship between smoking and IPF. This study revealed that the genetic predisposition to initiate smoking (based on 378 variants) and lifelong smoking (based on 126 variants) is associated with a higher risk of IPF. The results of this study also demonstrate the reliability of using MR to explore risk factors for IPF. Specific paper for reference is as follows:

1. Zhu J, Zhou D, Yu M, Li Y. Appraising the causal role of smoking in idiopathic pulmonary fibrosis: a Mendelian randomization study [published online ahead of print, 2023 May 22]. Thorax. 2023;thorax-2023-220012. doi:10.1136/thorax-2023-220012.

**Reviewer 2**

Dear Reviewer,

Thanks very much for taking your time to review this manuscript. I really appreciate your comments and suggestions! Please see below, for a point-by-point response to the reviewer’s comments and concerns. I hope that the changes I’ve made resolve all your concerns about the article. I’m more than happy to make any further changes that will improve the paper and facilitate successful publication.

**Point 1:** There are some issues regarding the methodology of this article. First of all instrumental variable is not explained adequately and some statistical terms are not used properly such as hypothesis instead of assumption or using of confusion instead of confounding.

**Response 1:** Based on your advice, we have corrected the misuse of statistical terms and provided a more detailed explanation of the concept of instrumental variables. Please see page 7, lines 73-82 for details.

**Point 2:** Moreover, the selection of the gene used for instrumental variable is not explained. I recommend to consult a methodologist to recheck this area.

**Response 2:** Indeed, confirming the genetic selection for instrumental variables can enhance the credibility of the chosen instrumental variables. However, based on the current GWAS data related to herpesvirus infection, we were unable to determine the necessary genes for instrumental variable selection. It is worth noting that the selection process for IVs in this paper was based on a substantial body of previously published Mendelian Randomization studies associating herpesvirus infection with diseases. These prior publications, to some extent, can substantiate the rationale and reliability of our instrumental variable selection. Specific papers for reference are as follows:

1. Zhang W, Wu P, Yin R, et al. Mendelian Randomization Analysis Suggests No Associations of Herpes Simplex Virus Infections With Multiple Sclerosis. Front Neurosci. 2022;16:817067. Published 2022 Mar 1. doi:10.3389/fnins.2022.817067.
2. Huang SY, Yang YX, Kuo K, et al. Herpesvirus infections and Alzheimer's disease: a Mendelian randomization study. Alzheimers Res Ther. 2021;13(1):158. Published 2021 Sep 24. doi:10.1186/s13195-021-00905-5.
3. Zhang Y, Qu J, Luo L, Xu Z, Zou X. Multigenomics Reveals the Causal Effect of Herpes Simplex Virus in Alzheimer's Disease: A Two-Sample Mendelian Randomization Study. Front Genet. 2022;12:773725. Published 2022 Jan 5. doi:10.3389/fgene.2021.773725.
4. Tan JS, Ren JM, Fan L, et al. Genetic Predisposition of Anti-Cytomegalovirus Immunoglobulin G Levels and the Risk of 9 Cardiovascular Diseases. Front Cell Infect Microbiol. 2022;12:884298. Published 2022 Jun 27. doi:10.3389/fcimb.2022.884298.
5. Zhu G, Zhou S, Xu Y, et al. Chickenpox and multiple sclerosis: A Mendelian randomization study. J Med Virol. 2023;95(1):e28315. doi:10.1002/jmv.28315.
