# Supplementary material for: Mendelian randomization reveals no correlations between herpesvirus infection and idiopathic pulmonary fibrosis
Source: PLoS One. 2023 Nov 28;18(11):e0295082. doi: 10.1371/journal.pone.0295082 (PMC10683991; doi:10.1371/journal.pone.0295082)
Supplement: Attachment — Submitted filename: Response to Reviewers.docx [file pone.0295082.s019.docx]

**Dear editor,**

Thank you for giving us the opportunity to submit a revised draft of the manuscript “Mendelian randomization reveals no correlations between herpesvirus infection and idiopathic pulmonary fibrosis” for publication in the Journal of PLOS ONE. We appreciate the time and effort that you and the reviewers dedicated to providing feedback on our manuscript and are grateful for the insightful comments on and valuable improvements to our paper. According to your suggestion, we revised the repetition in the article and revised the misquoted references. Specifically, we modify the following references

1. Widener, R. W. and Whitley, R. J. Herpes simplex virus. Handb Clin Neurol. 2014, 123,251-63.10.1016/b978-0-444-53488-0.00011-0.;

2. Epstein, M. A. Aspects of the EB virus. Adv Cancer Res. 1970, 13,383-411.10.1016/s0065-230x(08)60169-4; Zhang, L. J.; Hanff, P.; Rutherford, C.;

3. Churchill, W. H. and Crumpacker, C. S. Detection of human cytomegalovirus DNA, RNA, and antibody in normal donor blood. J Infect Dis. 1995, 171,1002-6.10.1093/infdis/171.4.1002.

to:

DUCKWORTH, H.J. LONGHURST, J.K. PAXTON, et al. "The Role of Herpes Viruses in Pulmonary Fibrosis," Front Med (Lausanne),8: 704222,2021.

I hope that the changes I’ve made resolve all your concerns about the article. I’m more than happy to make any further changes that will improve the paper and facilitate successful publication.

**Reviewer 1**

Dear Reviewer,

Thanks very much for taking your time to review this manuscript. I really appreciate your comments and suggestions! Please see below, for a point-by-point response to the reviewer’s comments and concerns. I hope that the changes I’ve made resolve all your concerns about the article. I’m more than happy to make any further changes that will improve the paper and facilitate successful publication.

**Point 1:** The observed weak correlation between the instrumental variables (IVs) and herpes infection merits a comprehensive discussion in the limitations section. It is vital to note that a strong correlation between IVs and exposure is a foundational assumption for Mendelian randomization (MR) studies.

**Respo**nse 1: We thank the reviewer for this constructive suggestion. According to your suggestion, we have had a comprehensive discussion in the limitation section of the ***Discussion***:

[***Discussion***: “First, our results need to be carefully interpreted. On one hand, one of the fundamental assumptions of MR is the strong correlation between IVs and exposure. In this study, despite using F-statistics to account for the influence of weak IVs, the association between the IVs and exposure may not be close because IVs were selected from different cohorts without clear overlap……Therefore, while our MR study provides preliminary evidence from a genetic perspective suggesting no causal relationship between herpesvirus infection and IPF, the possibility of such an association cannot be ruled out due to limitations in the strength of IVs and sample size. Further MR analysis will be needed when stronger IVs or GWAS with larger sample sizes become available.…”] (page 10-11, lines 281-294)

**Point 2:** It's crucial to acknowledge that conclusions derived from MR studies can vary significantly or even contradict each other. For instance, two MR studies examining the causative effects of smoking on Idiopathic Pulmonary Fibrosis (IPF) – Zhu et al., 2023 in Thorax, and Duckworth et al., 2020 in medRxiv – reached contrasting conclusions. Such differences could arise from the choice of IVs or the sample size of IPF patients. Highlighting these potential pitfalls is essential for reader awareness.

**Response 2:** Thank you for pointing out the problem and your valuable comments. We have highlighted this difference in the ***Discussion***. Since the MR study published by Duckworth et al. was not published in a journal included in SCI, we chose to use two MR studies on the association between gastroesophageal reflux disease and IPF with different conclusions to demonstrate this difference.

[***Discussion***: “On the other hand, conclusions drawn from MR studies can vary significantly. For instance, two MR studies examined the impact of gastro-esophageal reflux disease on IPF and arrived at completely different conclusions [44, 45]. This difference may be attributed to differences in the criteria for selecting IVs and the sample size of the exposure or outcome. Therefore, while our MR study provides preliminary evidence from a genetic perspective suggesting no causal relationship between herpesvirus infection and IPF, the possibility of such an association cannot be ruled out due to limitations in the strength of IVs and sample size. Further MR analysis will be needed when stronger IVs or GWAS with larger sample sizes become available……”] (page 10-11, lines 286-294)

**Point 3:** To summarize, while MR studies offer a novel perspective for exploring the causal link between herpesvirus infections and IPF through a genetic perspective, I urge a more cautious presentation of the conclusions. The absence of evidence for a causal relationship between an exposure and a disease, particularly when using a limited measure, doesn't negate the potential for such a relationship. This is especially pertinent when there is no positive control to validate the methodology employed.

**Response 3:** Thank you for your suggestion. At your request, we have revised the ***Discussion*** and ***Conclusions***.

[***Discussion***: “Therefore, while our MR study provides preliminary evidence from a genetic perspective suggesting no causal relationship between herpesvirus infection and IPF, the possibility of such an association cannot be ruled out due to limitations in the strength of IVs and sample size. Further MR analysis will be needed when stronger IVs or GWAS with larger sample sizes become available……”] (page 11, lines 290-294)

[***Conclusions***: “In conclusion, this study provides preliminary evidence that EBV, CMV, and HSV herpesviruses, and their related IgG levels, are not causally linked to IPF. However, our conclusions should be interpreted carefully. Further MR analysis will be necessary when stronger IVs and GWAS with larger sample sizes become available……”] (page 11, lines 307-310)
